# Targeting amino acid in tumor therapy

**DOI:** 10.3389/fonc.2025.1582116

**Published:** 2025-06-04

**Authors:** Mingpai Ge, Yuan Xu, Lu Cui, Enyuan Huang, Zhaorui Liu, Kai Yin

**Affiliations:** Department of Gastrointestinal Surgery, Changhai Hospital, Naval Medical University, Shanghai, China

**Keywords:** cancer metabolism, amino acid, metabolism, cancer therapy, amino acid metabolism

## Abstract

Tumor cells undergo profound metabolic reprogramming to sustain their rapid growth and proliferation, with amino acids serving as essential nutrients critical for protein synthesis, energy metabolism, nucleotide production, and redox balance. The increased reliance of tumor cells on specific amino acids represents a promising therapeutic target. This review provides an in-depth analysis of the biological roles of amino acids in cancer, identifies vulnerabilities associated with amino acid dependency, and discusses strategies to leverage these weaknesses for enhanced cancer treatment. We explore the mechanisms governing amino acid uptake, utilization, and metabolism in tumor cells, as well as their interactions with the tumor microenvironment. Additionally, the review addresses the challenges and prospects of targeting amino acid metabolism in cancer therapy, including issues of resistance, the complexity of metabolic pathways, and the potential for personalized treatment approaches.

## 1 Introduction

Tumor cells, unlike their normal counterparts, are characterized by a heightened demand for nutrient acquisition and utilization, driven by their need to sustain relentless growth and proliferation (1, 2). This metabolic reprogramming is marked by increased glycolysis, amino acid metabolism, and lipid biosynthesis (3–5). Amino acids, in particular, play indispensable roles in energy production, anabolic support, and the maintenance of cellular homeostasis, positioning amino acid metabolism as a pivotal focus in contemporary oncological research (6, 7). Targeting these metabolic dependencies offers significant therapeutic potential. Amino acid depletion therapy, which disrupts the availability and utilization of essential amino acids within tumor cells, presents a promising strategy for impairing tumor progression (8). In this review, we explore the feasibility and molecular mechanisms of targeting amino acids as potential therapeutic strategies in cancer treatment, and we also highlight the clinical applications of therapies targeting different amino acids.

## 2 Amino acid metabolism in cell

### 2.1 Amino acid in energy metabolism

**Figure 1:**
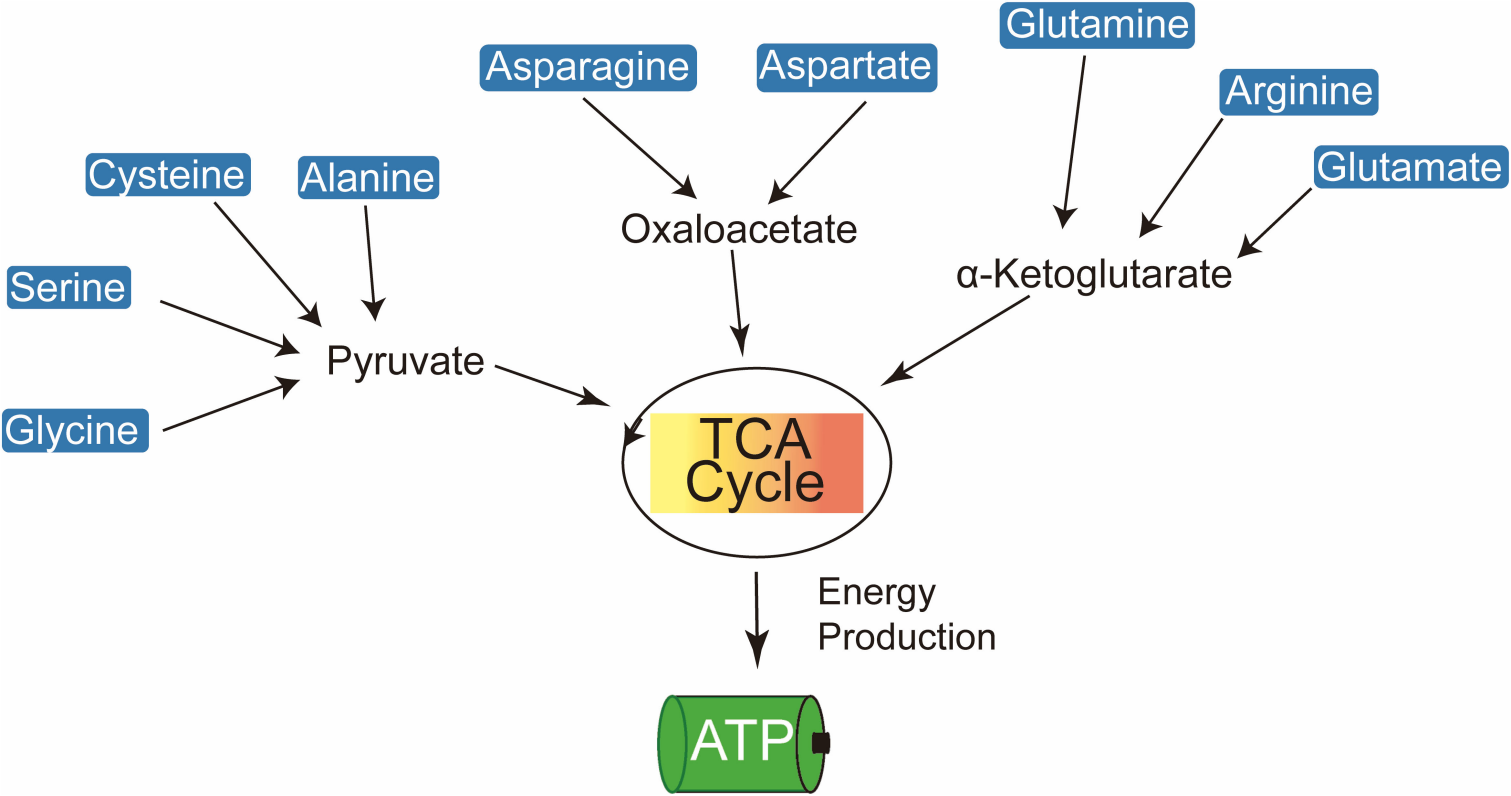
Amino acids contribute to cellular energy production by feeding into the TCA cycle and electron transport chain to generate ATP.

Amino acids are pivotal in generating metabolic intermediates, such as acetyl-CoA, which are critical for sustaining energy production through tricarboxylic acid cycle (TCA) (**Figure 1**) (9). In tumor cells, glutamine, a key amino acid, plays a central role in driving TCA via anaplerotic pathways, thereby supporting mitochondrial ATP synthesis (10–12). Glutamine undergoes conversion to α-ketoglutarate (α-KG), which is further metabolized into oxaloacetate (OAA), thereby fueling the TCA cycle through a series of enzymatic reactions in a process known as ‘glutaminolysis’ (13). Under conditions of glucose deprivation, there is a significant upregulation of glutamine-derived intermediates, such as fumarate, malate, and citrate, which further contribute to ATP production through the TCA cycle.

Elevated plasma concentrations of branched-chain amino acids (BCAAs) in pancreatic cancer patients serve as key metabolic substrates, particularly through their catabolism into acetyl-CoA, a critical intermediate in the TCA cycle (14). Among BCAAs, leucine is especially vital for the survival of melanoma cells. The hyperactivation of the RAS-MEK signaling pathway in melanoma leads to a pronounced dependence on leucine, with its deprivation resulting in impaired autophagy and subsequent apoptosis (15). Threonine also contributes to acetyl-CoA production, thereby sustaining TCA cycle activity. The catabolism of threonine, mediated by threonine dehydrogenase (TDH), produces glycine and acetyl-CoA, which are crucial for mitochondrial ATP production in mouse embryonic stem cells (16). Additionally, glutamine, a highly versatile amino acid, facilitates *de novo* amino acid synthesis through its conversion to glutamate, a reaction catalyzed by glutaminase (GLS) (17). Glutamate is further metabolized into α-KG via transamination, providing substrates for multiple metabolic pathways, including the synthesis of alanine, aspartate, and phosphoserine.

### 2.2 Amino acid in redox reactions

**Figure 2:**
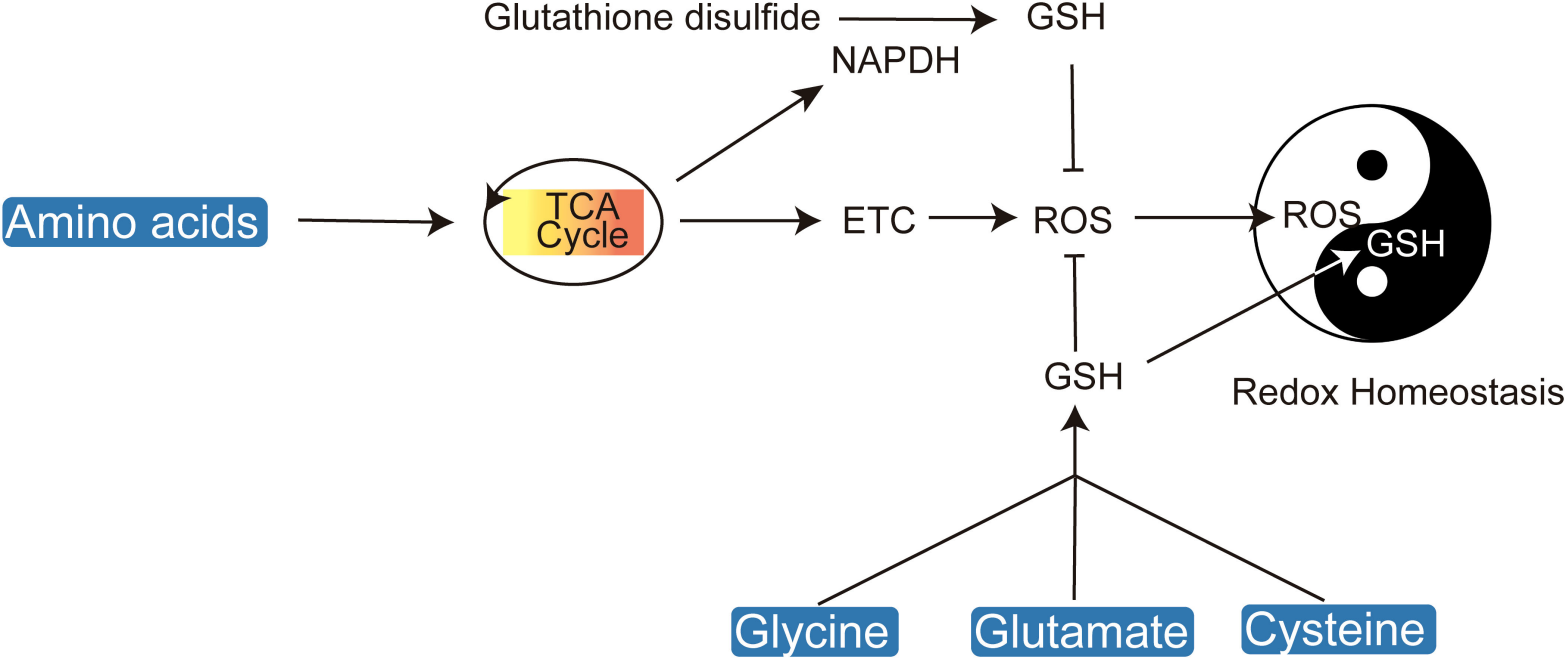
Amino acids play a crucial role in maintaining cellular redox homeostasis by regulating ROS and glutathione levels.

Cancer cells, characterized by their rapid proliferation, generate elevated levels of reactive oxygen species (ROS), which oxidize key macromolecules including lipids, proteins, and DNA, thereby inducing cellular damage (18–21). To counteract oxidative stress, amino acids play a crucial role in maintaining redox balance by supporting glutathione synthesis, thus protecting cells from ROS-induced apoptosis (**Figure 2**).

Antioxidant metabolites such as nicotinamide adenine dinucleotide phosphate (NADPH) and glutathione (GSH) in their reduced forms are essential for cellular redox homeostasis (22). Amino acids are critical precursors for GSH synthesis and NADPH production. The biosynthesis of glutathione necessitates the availability of glutamate, glycine, and cysteine, with cysteine being particularly crucial due to its redox-active thiol group (R-SH) (23, 24). Disruption of cysteine uptake exacerbates oxidative stress, leading ultimately to cell death (25). Cysteine can be directly transported into cells or imported in its oxidized form, cystine. The cystine/glutamate antiporter system xc- (xCT) facilitates the import of cystine, which is subsequently reduced to cysteine by GSH or thioredoxin reductase 1 (TRR1) (26). The rate-limiting step in glutathione synthesis is the ATP-dependent condensation of cysteine with glutamate, catalyzed by glutamate-cysteine ligase (GCL), leading to the formation of γ-glutamylcysteine, which is then conjugated with glycine to produce glutathione.

Serine serves as a critical intermediary in the folate-dependent biosynthesis of glutathione. The folate cycle’s intersection with the methionine cycle facilitates GSH production, and a deficiency in serine correlates with reduced GSH levels. This underscores the significance of serine synthesis as a glycolytic bypass essential for GSH replenishment (27, 28). Considering the pivotal role of amino acids in maintaining redox homeostasis, targeting the transport and intracellular synthesis pathways of cysteine, serine, glutamine, and glycine emerges as a promising approach for the development of novel redox-based therapeutics.

### 2.3 Amino acid in biosynthesis

**Figure 3:**
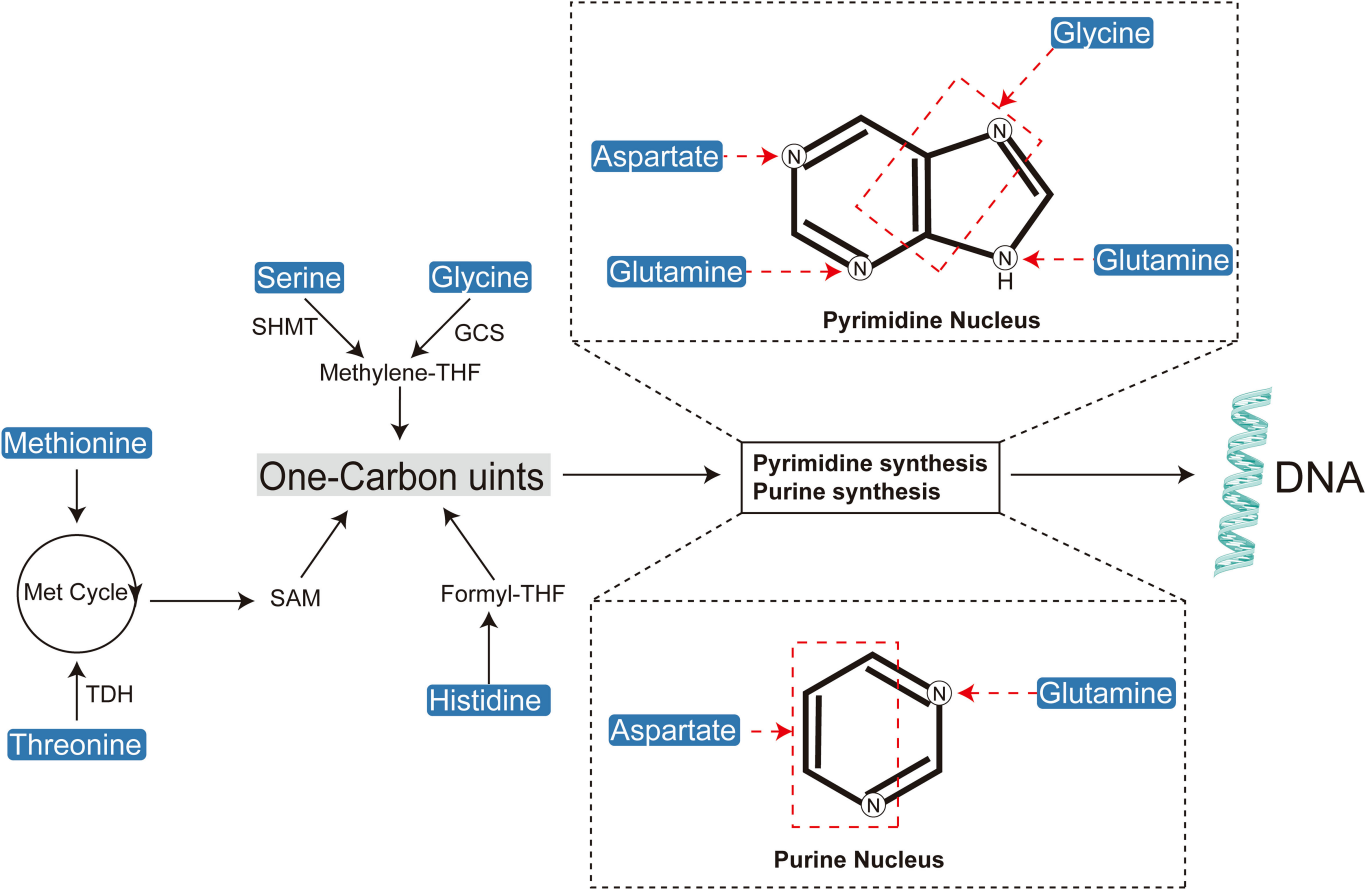
Amino acids play a crucial role in cellular biosynthesis by contributing to the synthesis of one-carbon units, purines, and pyrimidines.

A defining feature of tumor metabolic reprogramming is the enhanced biosynthetic capacity necessary to meet the macromolecular demands of DNA replication, cell division, and tumor proliferation (**Figure 3**) (29). Amino acids are indispensable precursors in nucleic acid synthesis, supplying both carbon and nitrogen atoms (30). Moreover, they play critical roles in supporting cellular growth and development by contributing to nucleotide and lipid biosynthesis (6, 31, 32). The biosynthesis of purine nucleotides is a complex process that requires formate, bicarbonate, and specific amino acids such as aspartate, glycine, and glutamine (33, 34). Glutamine and aspartate act as nitrogen donors for nucleobase formation and amino group addition, whereas glycine is incorporated directly into the purine ring or indirectly through the generation of one-carbon units (35, 36). Pyrimidine biosynthesis is relatively less complex, beginning with nucleobase formation and followed by condensation with phosphoribosyl pyrophosphate (PRPP) to produce the corresponding ribonucleotide (37, 38). The construction of the pyrimidine ring relies on glutamine, aspartate, and bicarbonate, with aspartate contributing both carbon and nitrogen atoms, and glutamine providing nitrogen for nucleobase formation and the addition of the cytidine amino group (39, 40).

Amino acids play a crucial role as carbon donors in lipid biosynthesis (41). Under hypoxic conditions, glutamine undergoes conversion to pyruvate, which subsequently enters the TCA cycle (42). This process supplies carbon substrates necessary for replenishing the acetyl-CoA pool that drives lipogenesis (43). Additionally, BCAAs contribute to lipogenesis through enhanced catabolic pathways, with approximately 30% of the acetyl-CoA used in lipid synthesis being derived from BCAAs (14).

One-carbon units are indispensable substrates for the *de novo* synthesis of purine and pyrimidine nucleotides. Several amino acids contribute to one-carbon metabolism, which ultimately supports DNA synthesis through the production of thymidine nucleotides (dTMP). Furthermore, one-carbon units are critical for the methylation of DNA, RNA, and proteins, facilitated by S-adenosylmethionine (SAM), a key methyl group donor (36, 44, 45).

Serine serves as a primary source of one-carbon units, converting to glycine while releasing a one-carbon unit in the process (46). Glycine can further contribute to one-carbon metabolism, while histidine, via the formation of N-formiminoglutamate (FIGLU), plays a significant role within the folate pathway (46–49). Methionine, once metabolized to SAM, is essential for various methylation reactions, including those involving DNA, proteins, and other biomolecules (50–52). Additionally, tryptophan metabolism, through the generation of N-formyl-5-hydroxykynurenine, provides formyl groups that are integral to one-carbon unit metabolism (53).

Arginine functions as a precursor for proline and a source of glutamate through its conversion mediated by 1-pyrroline-5-carboxylate (P5C) (54). The interconversion of serine and glycine is catalyzed by serine hydroxymethyltransferase (SHMT), a critical enzyme in folate-mediated one-carbon metabolism (55–57).

One-carbon metabolism, which includes the folate and methionine cycles, supplies essential methyl groups necessary for *de novo* nucleotide synthesis and DNA methylation (58). Tetrahydrofolate (THF) acts as a universal acceptor of one-carbon units, acquiring them during the conversion of serine to glycine within the folate cycle or methionine to homocysteine within the methionine cycle (59). The methionine cycle is intricately linked with a segment of the folate cycle, specifically involving the serine-glycine interconversion. Furthermore, methionine contributes to cysteine synthesis via the reverse transsulfuration pathway (60, 61). Cystathionine β-synthase (CBS) and cystathionine γ-lyase (CGL) catalyze the condensation of homocysteine with serine to form cystathionine, which is then converted to cysteine (62–64). The one-carbon unit generated during the serine-to-glycine conversion is crucial for thymidylate synthesis.

### 2.4 Amino acid in tumor therapy

#### 2.4.1 Glutamine

Glutamine is the most abundant amino acid in human plasma and plays a critical role in cancer cell proliferation (65). As the most rapidly consumed amino acid in cancer cells, its availability can profoundly influence tumor growth (66). Beyond its fundamental role in protein synthesis, glutamine is a precursor for α-KG, a pivotal intermediate in the TCA cycle. Additionally, glutamine is essential for the biosynthesis of all non-essential amino acids (NEAAs), with glutamate acting as an exchange factor for the import of essential amino acids (EAAs). Due to its high rate of consumption in cancer cells, glutamine is considered a conditionally essential amino acid and a potential rate-limiting factor in tumor expansion.

The metabolic pathways involving glutamine are tightly regulated in various cancers, with both *de novo* synthesis and degradation being upregulated by key oncogenes and tumor suppressors, including c-Myc and p53 (67). Tumors driven by oncogenes such as c-Myc or KRAS exhibit a marked dependence on exogenous glutamine, underscoring its potential as a therapeutic target and a promising biomarker for diagnostic imaging. The accumulation of radiolabeled glutamine in positron emission tomography (PET) scans has been effectively utilized for tumor detection (68).

Glutamine has emerged as a promising therapeutic target in oncology. Notably, CB-839 (Telaglenastat) has demonstrated encouraging anti-proliferative effects across various cancer types, including triple-negative breast cancer (TNBC), acute myeloid leukemia (AML), and non-small cell lung cancer (NSCLC), both *in vitro* and *in vivo (* 69, 70). Building on these promising preclinical results, CB-839 has progressed to Phase II clinical trials targeting both hematologic malignancies and solid tumors.

Cancer cells exhibit a heightened dependency on glutamine metabolism, facilitated not only through traditional uptake mechanisms—such as glutamine transporters and *de novo* synthesis—but also via alternative pathways including macropinocytosis and the proteolytic degradation of extracellular proteins (10, 71). A critical enzyme in glutamine catabolism, glutaminase, is essential for this metabolic process. Elevated extracellular cystine levels can enhance glutaminase activity by depleting intracellular glutamate, thereby increasing cancer cell reliance on glutaminase for glutamate replenishment. Tumors that overexpress xCT, a cystine/glutamate antiporter, are particularly dependent on glutaminase, making them prime candidates for glutaminase inhibition. Collectively, these findings highlight the significance of targeting glutamine metabolism in cancer therapy, with glutaminase inhibitors demonstrating substantial preclinical efficacy.

#### 2.4.2 Glutamate

Glutamate plays a central role in cancer cell metabolism despite its low plasma concentration and limited direct uptake (72). Intracellular glutamate is primarily generated from glutamine through the action of glutaminase, but it can also be synthesized from BCAAs and α-KG via branched-chain aminotransferases (BCAT1/2) (73–75). As a metabolic hub, glutamate is crucial for the biosynthesis of non-essential amino acids, including proline, aspartate, alanine, and serine, which serve as precursors for cysteine, glycine, asparagine, and arginine.

Glutamate dehydrogenase (GDH) catalyzes the conversion of glutamate to α-KG, releasing ammonia or transferring nitrogen to keto acids for the synthesis of NEAAs (76). Although glutamate contributes to TCA cycle anaplerosis, its nitrogen is preferentially channeled into NEAA biosynthesis in rapidly proliferating cancer cells, reflecting a nitrogen-sparing strategy. Inhibition of GDH has been shown to suppress tumor growth in certain cancers, underscoring its critical role in cancer metabolism (77). Notably, GDH can operate in reverse in some breast cancer cells, facilitating the fixation of ammonia nitrogen into glutamate.

The generation of NEAAs through transaminase-mediated processes from glutamine is essential for tumor growth across various cancer types. Additionally, glutamate is integral to the synthesis of the antioxidant glutathione, which is vital for cellular defense against oxidative stress. The complex interplay of glutamate sources and metabolic pathways in cancer cells poses challenges for therapeutic targeting, yet it also uncovers potential vulnerabilities (30, 78). Despite these challenges, glutamate metabolism remains a promising target for anticancer therapies, warranting further investigation.

#### 2.4.3 Asparagine

Asparaginase is a therapeutic enzyme widely used in the treatment of acute lymphoblastic leukemia (ALL) by depleting plasma asparagine (79, 80). ALL cells are particularly sensitive to asparagine depletion due to their low expression of asparagine synthetase (ASNS), the enzyme responsible for synthesizing asparagine from aspartate and glutamine-derived nitrogen (81). As a result, the reduction of serum asparagine levels leads to a critical shortage of this essential amino acid, impeding protein synthesis in ALL cells and ultimately resulting in their inhibition or eradication.

Despite the clinical success of asparaginase therapy, resistance remains a significant obstacle. This resistance often arises from the upregulation of ASNS expression within ALL cells, enhancing their ability to resynthesize asparagine. To address this challenge, researchers have developed ASNS inhibitors to overcome asparaginase resistance. Additionally, the role of asparagine in cancer cells extends beyond protein synthesis, acting as an exchange factor for other amino acids necessary for mTOR signaling activation (82). Lowered asparagine levels can modulate mTOR signaling, leading to reduced protein synthesis rates (83).

The role of asparagine in cancer cell growth and metastasis has increasingly drawn attention within the oncology research community. Recent studies underscore the critical importance of intracellular asparagine levels in promoting breast cancer metastasis, suggesting that therapeutic strategies such as asparaginase therapy, dietary asparagine restriction, or inhibition of asparagine synthetase may be effective against metastatic breast cancer. Asparagine also plays a central role in enhancing glutamine biosynthesis and facilitating the epithelial-mesenchymal transition (EMT), a process that is pivotal in cancer metastasis (84). Consequently, asparaginase (ASNase)-mediated depletion of asparagine inhibits both primary tumor growth and metastasis through mechanisms involving both asparagine deprivation and glutamine depletion (85).

Cancer cells subjected to hypoxia or mitochondrial dysfunction often exhibit altered glutamine metabolism. Under conditions of glutamine deprivation, asparagine becomes essential for preventing apoptosis, with citrate synthase (CS)—a positive regulator of apoptosis—serving as a key mediator in this process (86). Under normoxic conditions, CS facilitates the TCA cycle by catalyzing the condensation of glutamine-derived OAA with acetyl-CoA (87). However, silencing of CS redirects OAA from the TCA cycle towards aspartate and asparagine biosynthesis, thereby protecting cells from glutamine deprivation-induced apoptosis. Notably, exogenous asparagine fully restores cell viability in the absence of glutamine, whereas silencing asparagine synthetase induces apoptosis even in the presence of glutamine. These findings highlight the indispensable role of ASNS in tumor cell survival and progression. Indeed, high ASNS expression is associated with poor prognosis in brain tumors, including gliomas and neuroblastomas (88).

Non-malignant cells are generally capable of maintaining asparagine synthesis, which renders them relatively resistant to low serum asparagine levels compared to leukemia cells. Since the introduction of asparaginase therapy for acute lymphoblastic leukemia (ALL) in the 1960s, mortality rates in patients aged 0–24 years have significantly decreased. Targeting asparagine in cancer therapy thus holds considerable promise, particularly in ALL, where its efficacy is well-documented. Ongoing research and the development of novel therapeutic strategies, including asparagine synthetase inhibitors and other metabolic targets, have the potential to enhance treatment outcomes, overcome resistance, and extend the application of asparagine-targeted therapies to a broader range of malignancies.

#### 2.4.4 Aspartate

Aspartate metabolism plays a crucial role in cell proliferation and cancer progression (89). Aspartate is synthesized from OAA and glutamate via the enzyme aspartate aminotransferase and is widely distributed within both the cytoplasm and mitochondria (90). Beyond its metabolic functions, aspartate is integral to electron transfer between these cellular compartments through the malate-aspartate shuttle. Notably, aspartate production is markedly increased in the mitochondria of rapidly proliferating cells (91).

Aspartate is essential for cell viability, serving as a critical precursor for *de novo* purine and pyrimidine nucleotide synthesis. In certain cell types, it also contributes to biosynthesis and cell survival by supplying NADPH, which is necessary for neutralizing ROS (92).

The biosynthesis of aspartate is closely tied to the mitochondrial electron transport chain, which stimulates aspartate production in proliferative cells. This metabolic interdependence is highlighted by the ability to rescue electron transport chain-deficient cancer cells through the supplementation of exogenous aspartate. The pivotal role of aspartate in tumor growth is further evidenced *in vivo*, where the availability of aspartate restricts tumor expansion (93). As a result, targeting aspartate biosynthesis—particularly through the inhibition of aspartate aminotransferase—has emerged as a promising therapeutic strategy in cancer treatment.

#### 2.4.5 Arginine

Arginine is a multifunctional amino acid that plays a crucial role in protein synthesis and cellular metabolism (54, 94). As a key intermediate in the TCA cycle, arginine is essential for energy production. Additionally, arginine serves as a precursor for several critical biomolecules, including creatine, polyamines, and nitric oxide (NO) (95). Both polyamines and NO have been implicated in tumorigenesis, with NO contributing to oxidative stress and DNA damage.

The *de novo* biosynthesis of arginine, catalyzed by the enzymes argininosuccinate synthetase 1 (ASS1) and argininosuccinate lyase (ASL), is frequently dysregulated in cancer (96, 97). Increased expression of ASS1 and ASL is associated with poor prognosis in various malignancies, including glioblastoma, ovarian cancer, and gastric cancer. Conversely, reduced expression of ASS1 and ASL, leading to arginine deprivation, can trigger tumor cell death through mechanisms such as autophagy and apoptosis. Interestingly, normal cells can often survive arginine depletion by entering a quiescent state.

Given the dependency of certain tumors on exogenous arginine, therapeutic strategies targeting arginine metabolism have garnered attention. Arginase (ARG), which converts arginine to ornithine, has shown limited antitumor efficacy due to the potential activation of salvage pathways (98). In contrast, bacterial arginine deiminase (ADI), which catalyzes the conversion of arginine to citrulline and ammonia, has demonstrated potent antitumor activity in preclinical models and has shown promise in early-phase clinical trials (99, 100).

Arginine also plays a critical role in the urea cycle, primarily in the liver, where it detoxifies ammonia. Tumor cells often evade *de novo* arginine synthesis by silencing ASS1/ASL, thereby becoming dependent on exogenous arginine. This metabolic reprogramming facilitates tumor growth by redirecting nitrogen towards pyrimidine biosynthesis (101).

In conclusion, targeting arginine metabolism represents a compelling anticancer strategy. Arginine deprivation induces tumor cell death, and the inhibition of salvage pathways and the urea cycle presents further therapeutic opportunities (102). Agents such as ARG and ADI exemplify the potential of arginine-targeted therapies, with ongoing clinical trials assessing their efficacy and safety.

#### 2.4.6 Methionine

Methionine is an indispensable amino acid, crucial for protein biosynthesis and serving as a precursor for cysteine and polyamine synthesis (52, 103). Met also functions as the primary methyl donor for DNA, histone, and protein methylation, underscoring its importance in SAM production (104). The Met cycle, comprising a series of metabolic reactions, is often hyperactivated in tumor cells due to the upregulation of methionine adenosyltransferase 2A (MAT2A) (105). Unlike other amino acids, Met metabolism is intimately associated with malignant transformation. Downstream enzymes, such as nicotinamide N-methyltransferase (NNMT), contribute to the altered epigenetic landscape in cancer cells by depleting SAM and inhibiting DNA and histone methylation (106).

The Met salvage pathway, essential for Met replenishment independent of exogenous sources, involves enzymes such as methylthioadenosine phosphorylase (MTAP) and methionine synthase (MS), which are frequently downregulated in cancer cells (104, 107). The common co-deletion of MTAP with CDKN2A results in a complete reliance on exogenous Met in these cells. Increased Met uptake, as visualized through PET scans, has diagnostic and prognostic implications in high-grade gliomas, multiple myeloma, and brain tumors (108–111).

Given its pivotal role in protein synthesis and methylation, Met has emerged as a promising therapeutic target, particularly in tumors harboring mutations in epigenetic modifiers. Met-restricted diets have demonstrated substantial antitumor effects in mouse models of triple-negative breast cancer, colorectal cancer, sarcomas, gliomas, and mixed-lineage leukemia (MLL). Human studies have shown that long-term Met restriction is well-tolerated with minimal adverse effects.

Moreover, bacterial L-methionine-γ-lyase (METase) has demonstrated antitumor activity both *in vitro* and *in vivo* against neuroblastoma, colorectal cancer, melanoma, and brain tumors. METase catalyzes the conversion of Met into α-ketobutyrate, ammonia, and methanethiol, with limited toxicity observed in Phase I trials. Supplementation with homocysteine, vitamin B12, and folate may further enhance Met metabolism and utilization (58). In conclusion, Met represents a compelling therapeutic target in cancer, with Met restriction and metabolic enzyme-based strategies offering novel avenues for treatment.

#### 2.4.7 Serine

Serine metabolism has emerged as a critical aspect of cancer cell biology (48, 55). Cancer cells obtain serine either through uptake via specific transporters or through *de novo* synthesis from 3-phosphoglycerate, a glycolytic intermediate (112). The serine biosynthesis pathway, which involves the enzymes phosphoglycerate dehydrogenase (PHGDH), phosphoserine aminotransferase 1 (PSAT1), and phosphoserine phosphatase (PSPH), is frequently upregulated in malignancies such as triple-negative breast cancer and melanoma (113, 114). Within cancer cells, serine plays multiple roles, including contributions to purine biosynthesis, mitochondrial protein translation, lipid synthesis, and the regulation of glycolysis, as well as serving as a donor of one-carbon units.

Given serine’s pivotal role in tumorigenesis, targeting serine metabolism has become a focus in the development of anticancer therapies. Although PHGDH inhibitors have shown promise in preclinical models, the presence of exogenous serine in plasma often diminishes their effectiveness. It has been observed that serine biosynthesis becomes particularly critical in conditions of serine deprivation. Moreover, dietary restriction of serine and glycine has been explored as a potential therapeutic strategy. In murine models, reducing plasma serine levels through dietary interventions has been shown to inhibit tumor growth in a manner dependent on p53 and oxidative stress. However, the efficacy of this approach is modulated by a tumor’s intrinsic capacity for serine synthesis (115).

In summary, serine metabolism represents a complex and essential process in cancer progression. Therapeutic strategies targeting this pathway must take into account the specific tumor microenvironment and the dynamic interplay between serine biosynthesis and extracellular availability. A nuanced understanding of these factors is imperative for the successful development of serine metabolism-targeted cancer therapies.

#### 2.4.8 Glycine

Glycine, a multifaceted metabolite, plays a pivotal role in tumor metabolism (46, 116). It is predominantly synthesized from serine through the action of serine hydroxymethyltransferase (SHMT1 and SHMT2), contributing essential one-carbon units to the folate and methionine cycles (117–120). These metabolic pathways are fundamental for nucleotide biosynthesis, NADPH regeneration, redox homeostasis, protein translation, and epigenetic modifications, all of which are crucial processes for the rapid proliferation of cancer cells.

For decades, the inhibition of the folate cycle, as demonstrated by the use of methotrexate, has been a cornerstone in cancer therapy (121). Emerging evidence now indicates that histidine supplementation may enhance the efficacy of methotrexate (122). Additionally, targeting SHMT enzymes, which are central regulators of glycine metabolism, has shown promise as a novel anti-cancer strategy by disrupting one-carbon metabolism. The mechanisms underlying glycine uptake and utilization, including the glycine cleavage system, are also subjects of active investigation. A comprehensive understanding of glycine metabolism in cancer cells is vital for the development of innovative therapeutic strategies aimed at improving patient outcomes.

#### 2.4.9 Cysteine

Cysteine, a sulfur-containing amino acid, plays a crucial role in protein structure and function through the formation of disulfide bonds (123, 124). Beyond its structural importance, cysteine exhibits significant antioxidant properties, particularly in scavenging ROS, making it a key focus in cancer research (125).

Cysteine is primarily synthesized via the transsulfuration pathway, involving serine and methionine, but is predominantly acquired by tumor cells through the uptake of extracellular cysteine or its oxidized form, cystine (31, 126). The xCT antiporter facilitates the import of cystine, which is subsequently reduced intracellularly to cysteine. Notably, inhibition of xCT induces ferroptosis, a distinct form of cell death, highlighting xCT as a promising target for anticancer therapies.

Nuclear factor erythroid 2-related factor 2 (Nrf2) acts as a key transcriptional regulator of the light chain subunit of the xCT. Nrf2 enhances the initiation and elongation efficiency of xCT gene transcription, thereby leading to an upregulation in xCT protein synthesis levels. This transcriptional regulatory mechanism represents an important pathway for cells to enhance cystine uptake, promote glutathione synthesis, and thus respond to oxidative damage and maintain cellular redox homeostasis. Hence, the Keap1/Nrf2/xCT signaling pathway, a master regulator of cysteine metabolism, is frequently mutated in cancers and can be activated by oncogenic drivers such as KRas and PI3K, underscoring its critical role in tumorigenesis (127). Cysteine’s antioxidant function is further exemplified by its contribution to glutathione biosynthesis, a tripeptide that is notably enriched in cancer cells and is predominantly synthesized in response to Keap1/Nrf2/xCT activation induced by oncogenic mutations (128).

Paradoxically, despite elevated glutathione levels, many cancers exhibit resistance to inhibitors of glutathione synthesis, suggesting the existence of redundant antioxidant mechanisms. Nonetheless, targeting glutathione biosynthesis, particularly in combination with other therapies, remains a promising strategy. In conclusion, the multifaceted roles of cysteine in cancer, coupled with the intricate regulation of its metabolism, establish it as a compelling therapeutic target. Further elucidation of cysteine’s precise functions in cancer cells and the underlying regulatory mechanisms holds significant potential for the development of novel anticancer interventions.

#### 2.4.10 Proline

Proline metabolism plays a crucial role in tumorigenesis by influencing bioenergetics, osmotic balance, stress response, and apoptosis (129). Pancreatic cancer cells, in particular, exhibit significant metabolic plasticity, acquiring proline through biosynthesis, uptake, and collagen degradation via macropinocytosis under stress conditions (130). Paradoxically, despite the availability of multiple proline sources, protein synthesis in certain tumors can still be limited by proline availability, underscoring the potential of targeting proline metabolism for therapeutic intervention (131, 132).

The unique cyclic structure of proline confers diverse protein conformations, making proline-rich proteins vital for extracellular matrix integrity. These proteins modulate proline levels dynamically through biosynthesis and degradation pathways involving P5C synthase, pyrroline-5-carboxylate reductase, and proline dehydrogenase (PDH) (133). Notably, PDH-mediated proline catabolism is associated with tumor survival and metastasis, while MYC’s regulation of both proline synthesis and degradation highlights the oncogenic control over this metabolic pathway (134, 135).

The complex role of proline metabolism in tumor progression makes it a compelling target for therapeutic strategies. Modulation of proline metabolism has the potential to disrupt tumor cell metabolism, proliferation, and metastasis (136). Furthermore, combinatorial therapies that integrate proline metabolism inhibitors with conventional treatments may enhance anti-tumor efficacy. In conclusion, the critical role of proline metabolism in cancer warrants further exploration as a viable therapeutic strategy.

#### 2.4.11 Tyrosine

The precise biological functions of tyrosine within the tumor microenvironment remain less understood compared to other non-essential amino acids (137). However, its critical role in protein synthesis is well-established. Clinically, tyrosine metabolism has been leveraged for various applications, particularly in cancer management. Tyrosine-based positron emission tomography imaging has emerged as a valuable tool for tumor visualization and the assessment of therapeutic responses (138). These PET tracers take advantage of the commonly observed overexpression and hyperactivity of the amino acid transporter LAT1 in numerous tumor types. As a result, this imaging modality enhances diagnostic accuracy and facilitates treatment monitoring, thereby informing personalized cancer care. Beyond imaging, tyrosine analogs, such as SM-88, are currently under clinical evaluation for the treatment of multiple cancers (139).

Tyrosine’s influence on tumorigenesis extends beyond its role in protein synthesis, encompassing critical functions in signal transduction and antioxidant defense mechanisms (140). Its metabolic derivatives, including catecholamines, significantly impact cellular proliferation and survival. Emerging evidence suggests that these metabolites may modulate tumor behavior in specific cancer subtypes. The multifaceted involvement of tyrosine in these biological processes highlights its potential as a therapeutic target for novel cancer treatment strategies.

## Concluding remarks

The critical role and diverse functions of amino acids in tumor metabolism have been extensively studied, with increasing recognition of the importance of non-essential amino acids. While the roles of glutamine and glutamate are well-established, emerging evidence underscores the pivotal contributions of other NEAAs to cancer pathogenesis. Targeting NEAA metabolism presents a promising therapeutic strategy, with several novel interventions currently under clinical investigation. Additionally, dietary modulation of NEAA metabolism is being explored as a potential anticancer approach.

Targeting amino acids has emerged as a promising strategy in cancer therapy, offering a favorable toxicity profile compared to traditional treatments that induce DNA damage. However, the successful clinical application of this approach requires a comprehensive understanding of the metabolic dependencies of specific cancer types and their microenvironments to identify the most effective amino acid targets. The adaptive nature of tumor cells, which can evade amino acid deprivation through metabolic reprogramming, underscores the potential necessity for combination therapies. Such strategies could incorporate additional targeted agents to prevent resistance development, thereby disrupting multiple metabolic pathways within cancer cells and modulating the tumor microenvironment simultaneously. This multi-faceted approach has the potential to enhance both therapeutic efficacy and durability.

Over the past several decades, research has unequivocally established the pivotal role of amino acids in both promoting and suppressing tumorigenesis. These essential biomolecules not only serve as energy sources and building blocks for cancer cell growth but also yield metabolites with complex dual functionalities. For instance, nitric oxide (NO), derived from arginine, exhibits both pro-angiogenic and anti-tumor activities, including the upregulation of p53. Despite the availability of drugs targeting amino acid metabolism in the clinical setting, significant challenges remain. A deeper understanding of the metabolic plasticity and diversity in amino acid utilization by cancer cells could unveil novel therapeutic avenues, offering new strategies to combat cancer more effectively.
